# Randomized double-blind placebo-controlled multicenter trial for the effects of a polyherbal remedy, Yokukansan (YiganSan), in smokers with depressive tendencies

**DOI:** 10.1186/s12906-022-03788-7

**Published:** 2022-11-25

**Authors:** Maki Komiyama, Yuka Ozaki, Hiromichi Wada, Hajime Yamakage, Noriko Satoh-Asahara, Akihiro Yasoda, Yoichi Sunagawa, Tatsuya Morimoto, Shinji Tamaki, Masahiro Suzuki, Takuo Shibayama, Toru Kato, Yasumasa Okada, Toshiyuki Kita, Yuko Takahashi, Koji Hasegawa

**Affiliations:** 1 Clinical Research Institute, National Hospital Organization Kyoto Medical Center, 1-1 Mukaihata-Cho, Fukakusa, Fushimi-Ku, Kyoto, 612-8555 Japan; 2 Division of Molecular Medicine, School of Pharmaceutical Sciences, University of Shizuoka, Shizuoka, Japan; 3 National Hospital Organization NaraMedical Center, Nara, Japan; 4 Department of Clinical Research, National Hospital Organization Saitama Hospital, Saitama, Japan; 5 Department of Respiratory Medicine, National Hospital Organization OkayamaMedical Center, Okayama, Japan; 6 Department of Clinical Research, National Hospital Organization TochigiMedical Center, Tochigi, Japan; 7 Division of Internal Medicine and Laboratory of Electrophysiology, Murayama Medical Center, Tokyo, Japan; 8 Department of Respiratory Medicine, National Hospital OrganizationKanazawa Medical Center, Kanazawa, Japan

**Keywords:** Self-rating depression scale (SDS), Profile of moods states (POMS), Psychological effects

## Abstract

**Background:**

Smoking and depression are closely related and form a vicious cycle. Yokukansan (YiganSan) is a polyherbal remedy that has the effect of calming neuropsychiatric symptoms such as anger and irritation. To examine the efficacy of Yokukansan during smoking cessation (SC) therapy in smokers with depressive tendencies but without major depressive disorders requiring pharmacotherapy.

**Methods:**

A multicenter, double-blind, randomized, placebo-controlled, parallel-group comparison trial was conducted between June 2016 and May 2020 at 12 centers of the National Hospital Organization, Japan. This trial targeted smokers who first visited the SC outpatient clinics, did not receive any pharmacological treatment at the psychiatric or psychosomatic department, and scored 39 or more on the self-rating depression scale (SDS). Participants (*n* = 198) were randomly assigned to either the Yokukansan or placebo groups. The trial drug was initiated with the start of the SC treatment and continued for 12 weeks. The primary outcome was the high success rate of the SC treatment, and the secondary outcomes included changes in scores of the SDS and the Profile of Mood States (POMS) instrument.

**Results:**

The success rate of the SC treatment was similar between the placebo (63%) and Yokukansan (67%) groups (*P* = .649). The SDS scores (placebo: mean difference [MD] = -3.5, 95% confidence interval [CI][-5.8, -1.2], d = 0.42; Yokukansan: MD = -4.6, 95%CI[-6.8, -2.3], d = 0.55), and the “tension-anxiety” POMS-subscale scores (placebo: MD = -1.6, 95%CI[-2.5, -0.7], d = 0.52; Yokukansan: MD = -1.6, 95%CI[-2.9, -0.3], d = 0.36) showed significant improvement in both groups after the SC treatment. However, “depression-dejection” improved in the Yokukansan group (MD = -1.9, 95%CI[-3.1, -0.7], d = 0.44) but not in the placebo group (MD = -0.1, 95%CI[-1.0, 0.7], d = 0.04). Significant improvement in “fatigue” was noted in the Yokukansan group (MD = -2.1, 95%CI[-3.4, -0.9], d = 0.47) but not in the placebo group (MD = -0.5, 95%CI[-1.8, 0.8], d = 0.11). The time × group interaction on the improvement in “depression-dejection” was significant (*P* = .019).

**Conclusions:**

Yokukansan does not increase the SC treatment’s success rate but has additional positive effects on the psychological states due to the SC treatment in smokers with depressive tendencies but without apparent mental disorders.

**Trial registration:**

ID: UMIN000027036. Retrospectively registered at UMIN on April 18, 2017.

## Background

Reducing the risk of non-communicable diseases (NCDs) through proactive smoking cessation (SC) interventions is urgently required from economic and social perspectives. Given that a consensus has already been reached in the scientific community that smokers are nicotine-dependent patients, [1] the SC therapy is now provided to an ever-increasing number of smokers. In Japan, this therapy is eligible for the Japanese National Health Insurance scheme and has been provided as per the Standard Procedures for SC Therapy since 2006 [2].

Smoking is strongly associated with psychosocial stress, and several smokers have a depressive tendency, even if they have no apparent mental disorders [3]. This depressive tendency is the most serious factor that hinders successful SC treatment [4]. Therefore, it is imperative to comprehensively understand depressive tendency and nicotine dependency when providing smokers with SC interventions.

A traditional herbal medicine, Yokukansan (YiganSan), is indicated for neurosis and insomnia; it is also considered effective in reducing frustration, agitation, and aggressiveness [5–15]. Yokukansan is not only used in daily medical practice but is also available at Japanese pharmacies as an over-the-counter drug. Moreover, a placebo-controlled, double-blind, randomized trial found that the medication lowered agitation and aggression in a subset of patients with Alzheimer’s disease and lowered excitement and hostility in patients with treatment-resistant schizophrenia [9, 10]. Basic pharmacodynamic research has revealed that Yokukansan not only inhibits the release of glutamic acid [11], it also facilitates the uptake of glutamic acid [12] and the partial activation of serotonin receptors [13, 14].

Smokers with nicotine dependence suffer from chronic downregulation of nicotinic receptors, which leads to reduced serotonin release; [16] consequently, smokers tend to develop depression [17, 18]. Hence, Yokukansan, which performs a serotonin-releasing action [13, 14], may prevent depression in smokers. Additionally, it removes excessive glutamic acid, thereby reducing frustration, agitation, and aggressiveness [5–8, 11, 12]. A case study that used the Profile of Mood States (POMS) instrument reported that the use of Yokukansan for four weeks in five patients during the SC therapy tended to reduce anxiety, nervousness, and depression relative to pre-treatment levels [19]. However, no comparison of Yokukansan with control has been conducted.

Smokers often exhibit depressive tendencies even without major depressive disorders requiring pharmacotherapy [3]. Smoking and depression create a negative spiral, synergistically increasing the risk of NCDs. To maximize the effect of the SC treatment for reducing the risk of NCDs, it is essential to formulate a comprehensive treatment method for SC, including psychological care [4]. Hence, we designed a double-blind, randomized, controlled trial to investigate the effects and safety of a traditional herbal medicine, Yokukansan, during the SC therapy for smokers with depressive tendencies. The study aimed to examine whether Yokukansan could be useful in the SC therapy for smokers with latent depressive symptoms, not meriting psychiatric or psychosomatic treatment with antidepressants.

## Methods

### Subjects

Participants were current smokers who wanted to quit smoking. They were selected from among patients who had undergone initial examination but had not yet begun treatment at the SC outpatient departments at the National Hospital Organization (NHO) facilities or from among outpatients who had missed their SC treatment for at least one year after their last treatment. The inclusion criteria were as follows: (1) depressive tendency with a score between 39 and 59 on the self-rating depression scale (SDS) test [20–22] (questionnaire test for depression assessment); (2) nicotine dependence score of at least 5 on the Fagerstrom Test for Nicotine Dependence (FTND) [23–25]; (3) not currently undergoing pharmacotherapy at a department of psychiatry or taking psychosomatic medicine; and (4) age 20–80 years at the time of obtaining consent. Patients with a score of 53 or more on the SDS test or those clinically judged to require consultation by their attending physician were referred to a department of psychiatry/psychosomatic medicine to check whether they required pharmacotherapy. Patients were excluded from this study regardless of the SDS test score when psychiatry/psychosomatic physicians determined that they required drug administration. Patients were also excluded from this study based on a pre-trial examination and when their attending physician found that they met at least one of the following conditions: (1) administration of Yokukansan would be inadvisable based on the patient’s general condition (e.g., shock, current severe infectious disease, decompensated congestive heart failure, clinical findings that clearly indicated interstitial pneumonia, end-stage cancer, marked gastrointestinal weakening etc.); (2) complications, such as severe liver disease (Child–Pugh classification, stage C), end-stage kidney disease (creatinine clearance < 15 mL/min), and uncontrolled endocrine disease; (3) consistent use of herbal medicines containing licorice and glycyrrhizic acid; (4) history of Yokukansan allergy; (5) current symptoms of allergy to a medication; (6) current pregnancy, breast-feeding, or intention to become pregnant during the study period; (7) participation in this study would be inappropriate for any other reason (e.g., inability to understand the contents of the study because of dementia).

### Study design

This study was a multicenter, placebo-controlled, double-blind, randomized, parallel-group comparison trial. The study protocol was approved by an ethical committee (the NHO Headquarters Central Review Board) on May 13 2016 (H26-EBM(Intervention)-02). A total of 12 NHO facilities participated in this study. Before the start of the trial, the researcher in charge of statistics used a random number table to assign numbers to either Yokukansan or placebo and shared the results with the researcher in charge of group assignments, who assigned numbers alone to the boxes containing the packets of the test drug (Yokukansan or placebo). Thus, any other persons should not be able to know which one is which. The statistician was not involved in any enrollment, data entry, or data management of the clinical trial and was blinded.

The attending physicians provided detailed written explanations of the contents of this trial to all patients who fulfilled the inclusion criteria. Written informed consent was obtained from all participants. The study subject data were then entered into the electronic data capture system at the data center of the NHO headquarters, which handled patient enrollment in this trial. Immediately after enrollment, the electronic data capture system randomly assigned the subjects to the two groups (Yokukansan or placebo) in a 1:1 ratio using the dynamic allocation method, after which the attending physicians were informed of the number of trial drugs they had to administer to the patients. Based on the number indicated by the data center, the attending physicians administered the trial drugs without knowing whether they were administering Yokukansan or placebo. The group assignments were adjusted at the time of enrollment for confounding factors (age, sex, the FTND score, the SDS score, and drug use during the initial SC treatment) using the minimization method on dynamic allocations.

### Trial treatment protocol

The active study medication, Yokukansan (Tsumura Yokukansan Extract Granules for Ethical Use, TJ-54), [15] and placebo were purchased from Tsumura & Co. (Tokyo, Japan). Yokukansan 7.5 g contains dry extract 3.25 g of mixed crude drug of the following seven medicinal herbs: (1) *Atractylodes lancea* De Candolle (Compositae), (2) *Poria cocos* Wolf (Polyporaceae), (3) *Cnidium officinale* Makino (Umbelliferae), (4) *Uncaria rhynchophilla* Miquel (Rubiaceae), (5) *Angelica acutiloba* Kitagawa (Umbelliferae), (6) *Bupleurum falcatum* Linné (Umbelliferae), and (7) *Glycyrrhiza uralensis* Fisher (Leguminosae). This product also contains Japanese Pharmacopoeia Lactose Hydrate and Japanese Pharmacopoeia Magnesium Stearate. The placebo granules are composed of Japanese Pharmacopoeia Lactose Hydrate, Japanese Pharmacopoeia Corn Starch, Japanese Pharmacopoeia Dextrin, Japanese Pharmacopoeia Magnesium Stearate, Caramel, Yellow No. 4 Aluminum Lake, Iron Sesquioxide, Blue No. 1 Aluminum Lake, and identical in appearance.

With the start of the SC treatment based on the standard procedures, [2] administration of the trial drug (Yokukansan or placebo) was also initiated. This timing was designated as zero time point. The trial drugs were administered orally at a dose of one packet per administration, thrice per day (7.5 g/day), before breakfast, lunch, and dinner, or between meals, for 12 weeks. In subjects over 75 years of age and those with a bodyweight ≤ 50 kg, the dose was reduced to one packet per oral administration, twice per day (5.0 g/day), before breakfast and dinner, or between meals. Medication compliance was confirmed by recording the number of remaining packets for each subject at the end of the trial. The subjects were considered compliant if 70% or more of the prescribed doses had been administered.

Standard SC therapy has been performed as previously described [2–4]. The patients were treated with transdermal nicotine patches or oral varenicline, and examined on their first visit and two, four, eight, and twelve weeks after the first visit. On their repeated visits, maintenance of SC was checked, and specific advice regarding the continuation of SC was given by a nurse and a doctor.

### Assessment

The FTND is a world standard test used to assess the physical dependence on nicotine [20–22]. The following tests were conducted during the pre-administration screening and at the 12-week time point: the SDS test (test to measure depression) [4, 23–25] and the POMS-short form test (test of mood profile) [26, 27]. The subjects self-completed the test questionnaires as much as possible. If a subject failed to respond to an item or indicated clearly incorrect data, the subject was interviewed orally by a medical professional to confirm the data.

The SDS test has been employed in numerous clinical research studies on SC treatment and is reliable and valid [4, 23–25]. The POMS-short from test is commercially available, and the necessary parts were purchased for this study (Kanebo Shobo Co., Tokyo, Japan). It has six subscales: tension-anxiety (score range, 0–20), depression-dejection (score range, 0–17 for men, 0–19 for women), anger-hostility (score range, 0–18 for men, 0–19 for women), fatigue (score range, 0–20), vigor (score range, 0–20), and confusion (score range, 0–17 for men and 0–18 for women). The POMS-short form test is reliable and valid, and higher scores reflect higher levels of the construct being measured by the subscale [26, 27].

Each participant’s height, weight, and blood pressure were measured before taking Yokukansan or the placebo and again at the end of 12 weeks. They were also asked whether they had stopped smoking. Their exhaled carbon monoxide concentration was subsequently measured after the 12-week period by a nurse using the Pico-plus Smokerlyzer^R^ (Harada Cooperation, Osaka, Japan).

### Endpoints

The primary endpoint was the SC treatment’s success rate at the 12-week time point. This success rate (in %) was calculated by dividing the number of subjects who successfully stopped smoking by the total number of study subjects; “successful cessation of smoking” meant a subject had not smoked even a single cigarette over the past seven days and had an expiration CO concentration of 7 ppm or less.

The following were the two secondary endpoints: (1) the degree of change in the SDS and POMS scores from the pre-drug administration screening to the 12-week time point (assessment of post-SC depression), and (2) adverse events (AEs) during the trial drug administration period.

### Sample size calculation

In our previous study, the SC treatment’s success rate was 35% for patients with an SDS score of 39 or more; however, it was 69% for patients with an SDS score of less than 39 [4]. To determine the target sample size based on these data, we assumed a success rate of 35% for the placebo group and estimated that the success rate should increase by 12.5% for the Yokukansan group. Using a one-tailed significance level of 5% and a power of 80% yielded a target sample size of 210 cases in each group. Assuming 4%–5% ineligible cases, our final enrollment target for each of the two groups was 220, or a total of 440 cases.

### Statistical analysis

All statistical analyses were performed by a professional statistician using the Statistical Package for Social Sciences software program, version 24.0, for Windows (IBM Japan, Ltd., Tokyo, Japan). The normality of data was confirmed using the Shapiro–Wilk test. Continuous parametric data were expressed as the mean (standard deviation [SD]). Various baseline data were compared between the placebo and Yokukansan groups using Fisher’s exact probability test, the independent *t-*test, or the Mann–Whitney *U* test (Table 1). An intention-to-treat criterion was used to compare the rate of successful SC at the 12-week time point. AE frequencies between the two groups were compared using Fisher’s exact probability test (Table 2). For patients with good adherence to the trial drugs, clinical data, including the SDS and POMS scores (all scales) at the baseline and the 12-week time points, were compared using the paired-sample *t-*test in each treatment group separately (Tables 3 and 4). Additionally, concerning changes in data from the baseline to the 12-week time point, we analyzed the interaction between the placebo and Yokukansan groups using a two-way analysis of variance (Table 5). Statistical significance was set at *P* < 0.05.

**Table 1:** Baseline data of each enrolled patient group

	Placebo (*n* = 97)		Yokukansan (*n* = 94)		*P*-value	
Age (years)	59	(15)	61	(13)	.158	c
Female	*n* = 23	24%	*n* = 23	24%	> .999	a
Daily cigarette consumption					.796	b
< 15 pieces/day	*n* = 18	19%	*n* = 18	19%		
15–20 pieces/day	*n* = 49	51%	*n* = 42	45%		
21–30 pieces/day	*n* = 18	19%	*n* = 26	28%		
30 < pieces/day	*n* = 12	12%	*n* = 8	9%		
Duration of smoking (years)	38	(14)	39	(13)	.469	c
FTND score (points)	6.5	(1.5)	6.8	(1.6)	.220	c
Initial therapy for smoking cessation					.305	a
None, only counseling	*n* = 6	6%	*n* = 4	4%		
Varenicline	*n* = 55	57%	*n* = 45	48%		
Nicotine patch	*n* = 36	37%	*n* = 45	48%		
Past history Cancer	*n* = 14	14%	*n* = 14	15%	> .999	a
Myocardial infarction	*n* = 5	5%	*n* = 2	2%	.445	a
Stroke	*n* = 4	4.1%	*n* = 5	5%	.745	a
Diabetes mellitus	*n* = 12	12%	*n* = 7	7%	.335	a
Dyslipidemia	*n* = 9	9%	*n* = 14	15%	.271	a
Hypertension	*n* = 10	10%	*n* = 11	11%	.820	a
Consultation with psychiatrists	*n* = 2	2%	*n* = 2	2%	> .999	a
Anti-hypertensive agents	*n* = 30	31%	*n* = 30	32%	> .999	a
Anti-dyslipidemia agents	*n* = 15	15%	*n* = 12	13%	.680	a
Oral anti-diabetic agents	*n* = 10	10%	*n* = 10	11%	> .999	a
Insulin	*n* = 3	3%	*n* = 0	0%	.246	a
Sleeping pills	*n* = 5	5%	*n* = 5	5%	> .999	a
BMI (kg/m^2^)	23.2	(3.8)	23.2	(4.1)	.954	
SBP (mm Hg)	137	(23)	132	(20)	.123	
DBP (mm Hg)	79	(16)	77	(14)	.398	
Respiratory CO (ppm)	19	(14)	18	(12)	.466	
SDS test score	46.1	(4.4)	46.6	(5.1)	.419	
POMS score						
Tension-Anxiety(T-A)	5.6	(3.7)	5.5	(3.9)	.766	
Depression-Dejection(D)	3.6	(3.0)	4.4	(4.0)	.114	
Anger-Hostility (A-H)	4.0	(3.2)	3.9	(3.9)	.915	
Vitality(V)	5.0	(3.2)	5.4	(4.1)	.529	
Fatigue(F)	5.8	(4.3)	6.0	(5.0)	.790	
Confusion(C)	5.9	(2.8)	6.1	(3.5)	.646	

Data are presented as the mean (standard deviation)

*FTND score* Fagerström test for nicotine dependence score, *BMI* Body mass index, *SBP* Systolic blood pressures, *DBP* Diastolic blood pressures, *CO* Carbon monoxide, *SDS* test score, self-rating depression scale test score, *POMS* Profile of mood states

*p-*value: a, Fisher’s exact test; b, Mann–Whitney U test; c, unpaired *t-*test

**Table 2:** Mild or moderate adverse events

	Placebo (*n* = 97)		Yokukansan (*n* = 94)		*P*-value
Gastrointestinal symptoms	*n* = 11	11%	*n* = 11	12%	> .999
Infection	*n* = 1	1%	*n* = 3	3%	.363
Neurotic symptoms	*n* = 3	3%	*n* = 2	2%	> .999
Respiratory symptoms	*n* = 1	1%	*n* = 1	1%	> .999
Unidentified complaints	*n* = 1	1%	*n* = 2	2%	.617
Hypokalemia	*n* = 3	3%	*n* = 0	0%	.246
Liver damage	*n* = 1	1%	*n* = 0	0%	> .999

Gastrointestinal symptoms: nausea, vomiting, epigastralgia, constipation, diarrhea, abdominal fullness

Infection: common cold, bacterial pneumonia, otitis media, influenza

Neurotic symptoms: nightmares, headache, sleepiness

Respiratory symptoms: dyspnea, worsening of COPD

Unidentified symptoms: general malaise, palpitation, stiff shoulder

*p-*value: Fisher’s exact test

**Table 3:** Background data of each patient group for analysis of change in psychological states

	Placebo (*n* = 53)		Yokukansan (*n* = 55)		*P*-value	
Age (years)	60	(15)	62	(12)	.452	c
Female	*n* = 10	19%	*n* = 13	24%	> .641	a
Daily cigarette consumption					.841	b
< 15 pieces/day	*n* = 9	17%	*n* = 9	16%		
15–20 pieces/day	*n* = 25	47%	*n* = 26	47%		
21–30 pieces/day	*n* = 10	19%	*n* = 15	27%		
30 < pieces/day	*n* = 9	17%	*n* = 5	9%		
Duration of smoking (years)	39	(14)	40	(13)	.599	c
FTND score (points)	6.4	(1.5)	6.7	(1.6)	.302	c
Initial therapy for smoking cessation					.999	a
None, only counseling	*n* = 3	6%	*n* = 4	7%		
Varenicline	*n* = 32	60%	*n* = 32	58%		
Nicotine patch	*n* = 18	34%	*n* = 19	34%		

Data are presented as the mean (standard deviation)

*FTND score* Fagerström test for nicotine dependence score

*p-*value: a, Fisher’s exact test; b, Mann–Whitney U test; c, unpaired *t-*test

**Table 4:** Patient data before and after 12 weeks of smoking cessation intervention

	Placebo group (*n* = 53)		*p* value ^a^ Baseline vs. 12-week	Yokukansan group (*n* = 55)		*p* value ^a^ Baseline vs. 12-week
	Baseline	12-week		Baseline	12-week	
BMI (kg/m^2^)	22.7 (3.4)	23.1 (3.6)	***.009***	23.5 (3.7)	24.0 (3.8)	** < *.001***
SBP (mm Hg)	138 (26)	137 (21)	*.968*	131 (18)	137 (18)	***.018***
DBP (mm Hg)	78 (19)	77 (15)	*.454*	76 (13)	78 (13)	*.313*
Respiratory CO (ppm)	18.9 (10.8)	4.8 (3.9)	** < *.001***	18.4 (10.5)	4.9 (5.6)	** < *.001***
SDS test score	45.6 (4.2)	42.1 (8.3)	***.004***	46.8 (5.2)	42.3 (7.4)	** < *.001***
POMS score						
Tension-Anxiety(T-A)	5.4 (3.5)	3.8 (3.4)	***.001***	5.3 (3.7)	3.7 (3.5)	***.013***
Depression-Dejection(D)	3.4 (3.2)	3.3 (3.0)	*.768*	4.4 (4.2)	2.6 (3.2)	***.003***
Anger-Hostility (A-H)	3.8 (3.3)	3.7 (3.8)	*.890*	4.4 (4.5)	3.1 (4.3)	*.062*
Vitality(V)	5.3 (3.7)	6.5 (4.3)	*.053*	5.3 (4.2)	6.5 (5.0)	*.123*
Fatigue(F)	5.7 (4.5)	5.2 (4.6)	*.452*	6.2 (4.8)	4.1 (4.0)	***.002***
Confusion(C)	5.7 (3.0)	5.1 (3.0)	*.174*	5.8 (3.1)	4.8 (2.5)	***.015***

Data are presented as the mean (standard deviation)

*BMI* Body mass index, *SBP* Systolic blood pressures, *DBP* Diastolic blood pressures, *CO* Carbon monoxide, *SDS* test score, self-rating depression scale test score, *POMS* Profile of mood states

^a^ paired t test between baseline and 12-week

**Table 5:** Interaction between placebo and yokukansan groups on change after 12 weeks of smoking cessation intervention

Placebo group (*n* = 53)					Yokukansan group (*n* = 55)				interaction [baseline and 12-week]x [Placebo and Yokukansan]	
	Mean difference	95% Confident interval		d ^a^	Mean difference	95% Confident interval		d ^a^	*P* value ^b^	pη2 ^c^
BMI (kg/m^2^)	0.45	0.12,	0.17	0.382	0.52	0.29,	0.76	0.616	.705	0.002
SBP (mm Hg)	-0.12	-5.92,	5.68	0.006	5.87	1.07,	10.7	0.337	.112	0.026
DBP (mm Hg)	-1.41	-5.17,	2.35	0.106	1.70	-.165,	5.04	0.140	.217	0.012
CO (ppm)	-14.1	-17.2,	-11.0	1.369	-13.5	-16.9,	-10.0	1.131	.781	0.014
SDS test score	-3.51	-5.83,	-1.19	0.417	-4.56	-6.81,	-2.30	0.551	.518	0.000
POMS score										
*Tension-Anxiety(T-A)*	-1.59	-2.48,	-0.71	0.518	-1.60	-2.85,	-0.35	0.363	.992	0.001
*Depression-Dejection(D)*	-0.12	-0.95,	0.71	0.042	-1.88	-3.11,	-0.65	0.436	**.019**	0.024
*Anger-Hostility (A-H)*	-0.08	-1.26,	1.10	0.020	-1.26	-2.59,	0.07	0.270	.186	0.001
*Vitality(V)*	1.16	-0.02,	2.34	0.283	1.14	-0.32,	2.60	0.222	.980	0.000
*Fatigue(F)*	-0.49	-1.79,	0.81	0.108	-2.14	-3.43,	-0.85	0.472	.073	0.006
*Confusion(C)*	-0.57	-1.40,	0.26	0.197	-1.00	-1.80,	-0.20	0.356	.457	0.002

Data are presented as the mean ± standard deviation

*BMI* Body mass index, *SBP* Systolic blood pressures, *DBP* Diastolic blood pressures, *CO* Carbon monoxide, *SDS* Test score, self-rating depression scale test score, *POMS* mood state profile

^a^ effect size difference (Cohen d)

^b^ two-way analysis of variance to determine the interaction between time (baseline and 12-week) and group (placebo group and Yokukansan group)

^c^ effect size for interaction (Partial eta squared)

## Results

### Success rate of smoking cessation

The participants (*n* = 198) were randomly assigned to one of the two groups: one that received the placebo and another that received Yokukansan, during the registration period between June 2016 and May 2019. The NHO Headquarters Central Review Board decided to terminate enrollment for the trial after three years based on time- and funding-related considerations. After excluding seven ineligible cases, the data of the remaining 191 patients (145 men and 46 women) were analyzed. The baseline data for each group are presented in Table 1. No significant differences were observed between the placebo and Yokukansan groups regarding age, sex, number of cigarettes per day, years of smoking, the FTND score, drugs used for the initial SC treatment, medical history, oral medication, and psychological states. The SC success rate at the 12-week time point was similar for both groups: 63% for the placebo group and 67% for the Yokukansan group (*P* = 0.649).

### Adverse events

Two subjects experienced severe AEs: Case 1, pneumothorax, and Case 2, eruption with fever, both in the placebo group (2.1%, 2/97). Both the cases were judged to be unrelated to trial drug administration. Table 2 shows the specific symptoms and findings of mild and moderate AEs and their frequencies. No AEs specific to Yokukansan were observed.

### Change of SDS and POMS subscale scores

**Fig. 1:**
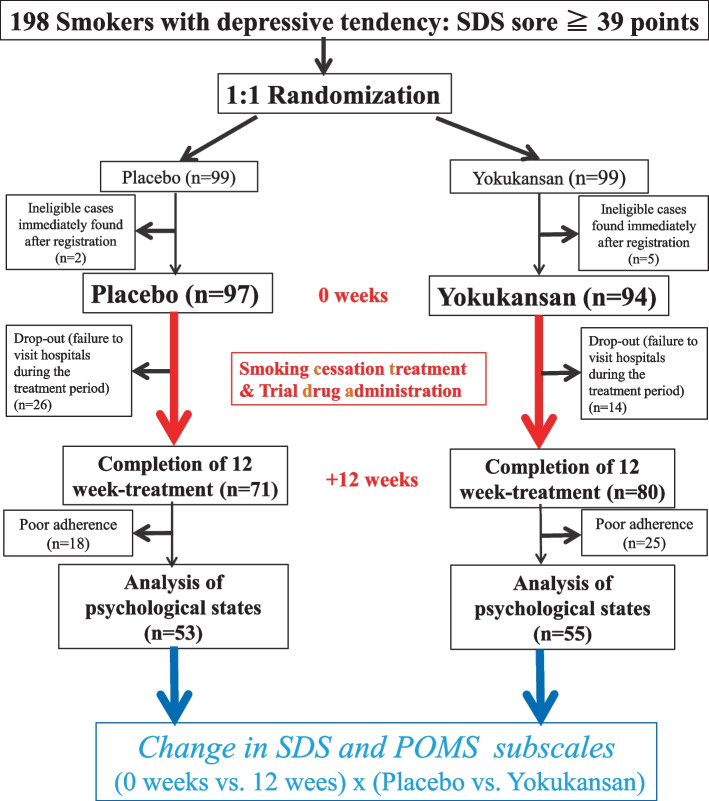
CONSORT Diagram

The percentage of subjects who completed the 12-week administration protocol was 73% (71/97) in the placebo group and 85% (80/94) in the Yokukansan group (Fig. 1). The percentage of subjects who adhered well to the investigational drug (i.e., those who took 70% or more of the prescribed doses) was 75% (53/71) in the placebo group and 69% (55/80) in the Yokukansan group. The background data of these 108 participants are presented in Table 3. Data at baseline and at the 12-week time point are presented in Table 4. The body mass index significantly increased by approximately the same amount. Further, the exhaled CO concentration levels markedly decreased in both groups. There was a slight increase in systolic blood pressure in the Yokukansan group, but no change was observed in the placebo group. The SDS data were missing for one patient in the Yokukansan group; the POMS data were missing for four patients in the placebo group and five patients in the Yokukansan group—the remaining data were analyzed. The SDS scores improved significantly after the SC treatment in the placebo (*P* = 0.004) and Yokukansan groups (*P* < 0.001). The tension-anxiety scores also showed significant improvement in the placebo (*P* = 0.001) and Yokukansan groups (*P* = 0.013) after the SC treatment. In contrast, the depression-dejection (*P* = 0.003), fatigue (*P* = 0.002), and confusion (*P* = 0.015) POMS scores showed significant improvement only in the Yokukansan group. Differences in data between the baseline and the 12-week time points are presented in Table 5. The two-way analysis of variance revealed that depression-dejection exhibited significantly greater improvement in the Yokukansan group than in the placebo group (*P* = 0.019).

## Discussion

In Japan, Yokukansan is given for neurosis and insomnia in general outpatient clinics [15]. This study examined the efficacy and safety of Yokukansan in combination with the standard SC treatment in smokers with depressive tendencies in a multicenter, placebo-controlled, randomized, parallel trial. The participants did not receive standard psychiatric/psychosomatic medications. The frequency of AEs was similar between the groups, and no AEs specific to the Yokukansan group were observed. Consequently, the safety of the concomitant use of Yokukansan in the standard SC treatment was confirmed within the context of this trial.

Although the antidepressant bupropion is an effective aid for SC, selective serotonin reuptake inhibitors have not been found useful [28]. The neurotransmitters that are temporarily deficient due to SC include serotonin, noradrenaline, and dopamine. Varenicline, a partial nicotinic agonist, induces SC via dopamine release. In this study, successful SC was defined as having not smoked any cigarette in the past week and an exhaled CO concentration of 7 ppm or less. We examined short-term (12 weeks) SC success rates, and the partial agonist of serotonin receptors [6, 13, 14], Yokukansan, did not increase SC success rates when used in combination with standard SC treatment.

There was a slight increase in systolic blood pressure in the Yokukansan group, but no change was observed in the placebo group. The reason of the significant increase in SBP observed in the Yokukansan group is unclear. *Glycyrrhiza uralensis* a component contained in Yokukansan, is one of Licorice, which is known to cause pseudo-aldosteronism due to the inhibition of type 2 11β-hydrosteroid dehydrogenase [29]. We cannot rule out the possibility that pseudo-aldosteronism may have more or less caused the observed SBP increase in the Yokukansan group. We believe that blood pressure should be monitored in long-term use of Yokukansan.

The exhaled CO concentration showed a marked decrease, indicating that the number of cigarettes smoked per day was significantly reduced during the SC treatment period, even among those who failed to cease smoking. The SDS and tension-anxiety—a POMS parameter—scores significantly improved after the SC treatment in both groups relative to pre-treatment levels. A previous study showed that the SDS scores significantly improved 12 weeks after the start of the SC treatment in successful quitters, [23] which is consistent with our current results. Although several reports indicate that SC is important for mental health from 1 to 9 years of period, [30, 31] the results of our study demonstrate that even short-term SC (12 weeks) significantly improves individuals’ psychological state.

The depression-dejection POMS parameter improved in the Yokukansan group but not in the placebo group, and a significant difference was observed in the degree of improvement between the groups. Pharmacological studies in rodents have shown evidence of serotonin 2A receptor downregulation action [13] and serotonin 1A receptor stimulating action of Yokukansan [14]. These pharmacological effects may, in part, explain the improvements observed in this study. However, as Yokukansan performs multiple pharmacological actions, other mechanisms involved in improving these symptoms should be further investigated.

The normal range of the SDS test score was 35 ± 8 points, and that of patients with depression was 60 ± 7 points [20]. The SDS score in our study population was 46 ± 5 points and intermediate between the normal and depression groups. The present study targeted patients on the border between normal and major depressions, who were judged not to require antidepressants. In humans, Yokukansan has been shown to reduce agitation and aggression in a subset of patients with Alzheimer disease, [32] and reduce excitement [33] and hostility in patients with treatment-resistant schizophrenia [10]. However, to date, there have been no reports of double-blind, randomized, controlled trials examining the effects of Yokukansan in smokers. Smokers are 2.25-times more likely to be depressed and 4.3-times more likely to commit suicide, [34, 35] than non-smokers. The cessation of smoking engenders a host of positive effects on common psychological issues in smokers; indeed, it improves their mental health, generates positive emotions, and reduces depression, anxiety, and stress [30, 31]. However, smokers prone to depression are more likely to resume smoking even after quitting [36]. While individuals who quit smoking face a lower risk of suicide than smokers, they also tend to have a relatively higher risk than non-smokers [35, 37]. This study showed, for the first time, that the use of Yokukansan along with the SC treatment further improved psychological symptoms in smokers with latent depression who did not benefit from psychiatric or psychosomatic treatment.

There are several limitations. First, the details of why Yokukansan did not lead to an increase in short-term SC success rates are unknown. Second, the number of subjects did not reach the scheduled target number of cases (n = 440). Third, this study examined short-term (12 weeks) SC success rates. Long-term interventional studies are needed to determine whether Yokukansan could lead to improvement in the SC maintenance in future.

## Conclusions

Concomitant use of Yokukansan during the SC therapy does not increase the therapy’s success rate in smokers with depressive tendencies but without apparent mental disorders. The SC therapy significantly improves smokers’ psychological states, and an additional Yokukansan use further improves some depressive symptoms.

## Data Availability

The datasets used and/or analysed during the current study are available from the corresponding author on reasonable request.

## Abbreviations

SC: Smoking cessation
SDS: Self-rating depression scale
POMS: Profile of mood states
MD: Mean difference
CI: Confidence interval
NCDs: Non-communicable diseases
NHO: National hospital organization
FTND: Fagerstrom test for nicotine dependence
SD: Standard deviation
